# Geometric phase-encoded stimuli-responsive cholesteric liquid crystals for visualizing real-time remote monitoring: humidity sensing as a proof of concept

**DOI:** 10.1038/s41377-023-01360-7

**Published:** 2024-01-24

**Authors:** Shi-Long Li, Zhao-Yi Chen, Peng Chen, Wei Hu, Chaohong Huang, Sen-Sen Li, Xuejia Hu, Yan-Qing Lu, Lu-Jian Chen

**Affiliations:** 1 Department of Electronic Engineering, School of Electronic Science and Engineering, Xiamen University, 361005 Xiamen, China; 2 College of Engineering and Applied Sciences, Nanjing University, 210093 Nanjing, China; 3 Fujian Key Laboratory of Ultrafast Laser Technology and Applications, Xiamen University, 361005 Xiamen, China

**Keywords:** Liquid crystals, Optical sensors

## Abstract

Liquid crystals are a vital component of modern photonics, and recent studies have demonstrated the exceptional sensing properties of stimuli-responsive cholesteric liquid crystals. However, existing cholesteric liquid crystal-based sensors often rely on the naked eye perceptibility of structural color or the measurement of wavelength changes by spectrometric tools, which limits their practical applications. Therefore, developing a platform that produces recognizable sensing signals is critical. In this study, we present a visual sensing platform based on geometric phase encoding of stimuli-responsive cholesteric liquid crystal polymers that generates real-time visual patterns, rather than frequency changes. To demonstrate this platform’s effectiveness, we used a humidity-responsive cholesteric liquid crystal polymer film encoded with a *q*-plate pattern, which revealed that humidity causes a shape change in the vortex beam reflected from the encoded cholesteric liquid crystal polymers. Moreover, we developed a prototype platform towards remote humidity monitoring benefiting from the high directionality and long-range transmission properties of laser beams carrying orbital angular momentum. Our approach provides a novel sensing platform for cholesteric liquid crystals-based sensors that offers promising practical applications. The ability to generate recognizable sensing signals through visual patterns offers a new level of practicality in the sensing field with stimuli-responsive cholesteric liquid crystals. This platform might have significant implications for a broad readership and will be of interest to researchers working in the field of photonics and sensing technology.

## Abstract (web summary)

The proposed visual remote sensing platform utilizes geometric phase encoding of stimuli-responsive cholesteric liquid crystal polymers to generate intuitive image signals, showcasing its proof of concept by real-time humidity monitoring.

## Graphical abstract

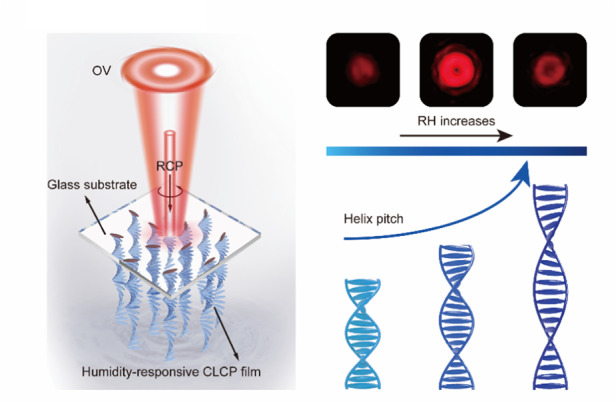

## Introduction

Sensors have become increasingly essential in many fields, including biology, medicine, the chemical industry, communication, and aerospace, as the demand for environmental safety detection, rapid information acquisition, and high-precision data processing continues to grow. In response to this demand, scientists are seeking to develop fast, user-friendly, and affordable sensing technology across various applications. One important area of research is the development of sensing materials that enable detection through visual analysis. Stimuli-responsive photonic crystals (PCs) with complex periodic microstructures are highly appealing for optical sensors, as they can produce structural colors without requiring batteries^1–3^. Among the stimuli-responsive PCs, cholesteric liquid crystals (CLCs) have attracted considerable attention due to their self-assembled helical superstructures and easy-to-fabricate one-dimensional PC structures^4–6^. The helical pitch of CLCs can be tuned to produce vividly colored Bragg reflections over the entire visible wavelength range, which can be observed with the naked eye. The reflection band, in the case of normal incidence, can be determined as *n*_o_*p* < *λ* < *n*_e_*p*, where *n*_o_ and *n*_e_ are the ordinary and extraordinary refractive indices and *p* is the helix pitch of CLC.

Previous research has underscored the responsiveness of CLC to external stimuli, endowing them with performance tunability^7–10^. Consequently, CLC emerges as a viable candidate for fabricating diverse optical sensors. Literature reports multiple types of CLC-based optical sensing materials, inclusive of responsive substance-doped CLC and CLC polymer films^11–13^. These sensors demonstrate an adaptability to environmental changes such as variations in light^14^, temperature^15^, humidity^16,17^, strain^18^, and pH^19^, or in response to chemical analytes including organic vapor^20,21^, alcohols^22^, amines^23^, and metal ions^24,25^. Despite the advantage of producing visible signals through structural color changes, the precise measurement of reflected wavelengths remains reliant on expensive equipment like spectrometers. This limitation signifies a critical hurdle for CLC optical sensors, warranting further research to overcome it.

Along with the emerging demand for planar optical elements (POEs) as flat and miniaturized optical devices, impressive progress has been achieved with the geometric phase (also known as the Pancharatnam–Berry phase)^26^ originated from the spin–orbit interaction (SOI) of light. The geometric phase is strictly related to the space transformation of the polarization state of light in an anisotropic medium like liquid crystals (LCs). In 2016, spin–orbit coupling-induced geometric phases were discovered in circularly polarized Bragg-reflected light of planar CLCs^27–29^. In recent years, efforts have focused on imbuing the geometric phase into the reflected light within the photonic band gap (PBG) by manipulating the local orientation of the CLC helical superstructure, leading to diverse applications in photonics^30–34^. By taking advantage of space-variant stimuli-responsive chiral superstructures, active POEs such as deflectors^35,36^, lenses^28,37^, Airy beams^38^, and optical vortex (OV) generators^34,39^ that operate in polychromatic working bands have been developed.

Geometric phase encoding within planar CLC devices exhibits compelling properties, notably the capacity to vary its reflected diffracted light field corresponding to the reflected wavelength band^30,40^. This adaptation yields an image-based stream of extensive sensory data, thereby extending the potential of conventional Bragg PBG sensing techniques, which operate on wavelength or frequency parameters^41^. OV introduces a unique degree of freedom (DoF), namely orbital angular momentum (OAM). Among these, Vortex Beams (VBs) serves as an exemplary representation and have emerged as a fundamental tool for exploring wavelength and OAM tunable OVs. Characterized by a consistent angular momentum and helical phase structure, VBs exhibit remarkable stability during transmission. This stability is primarily attributed to the VB’s capacity to retain angular momentum, rendering it significantly more stable in comparison to conventional beams^42^. Furthermore, in some situations, such as hazardous chemical environments, high-altitude weather surveillance, and aerospace conditions, where traditional optical sensors can face difficulties^43,44^. However, by employing CLC sensors integrated with geometric phase (like *q*-plate, *q* is the topologic charge) encoding and augmented with highly directional lasers, real-time, long-distance sensing can be accomplished, effectively circumventing the restrictions inherent in conventional sensors.

**Fig. 1:**
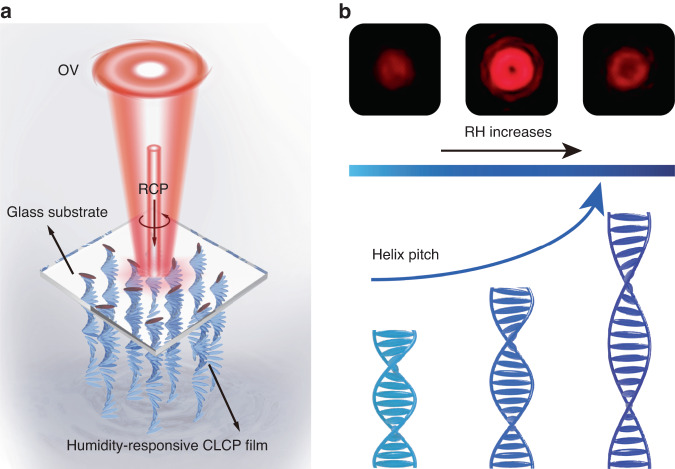
The schematic illustration of the humidity sensor platform and its operational principle. **a** Schematic of the proposed humidity-responsive CLCP film-mediated humidity sensor platform. **b** The alteration of RH induces a change in the CLCP helix pitch and consequently generates a corresponding variation in the morphology of VB

In this study, we present a visual sensing platform that utilizes geometric phase encoding of stimuli-responsive CLC polymer (CLCP) to provide real-time sensing information through the generation of visual patterns, eliminating the need for frequency changes. To validate the efficacy of the platform, we used a humidity-responsive CLCP film encoded with *q*-plates and provide real-time feedback using the OVs in Fig. 1a. Variations in relative humidity (RH) induce modifications in the CLCP helix pitch, consequently resulting in alterations of the VBs reflected by the film (Fig. 1b). Furthermore, we employed two schemes to extend the range of humidity monitoring and enable real-time capture of humidity levels: a CLCP film encoded with a four-quadrant *q*-plate array pattern and a dual-wavelength humidity monitoring system. Significantly, the platform includes a remote sensing feature, attributed to the high directionality and extensive transmission range of laser beams. These results significantly broaden the technical application area of CLCPs as broadband reflective POEs while highlighting the potential of stimuli-responsive geometric phase as a promising sensing mechanism.

## Results

### Fabrication of stimuli-responsive CLCP films encoded with *q*-plate patterns for humidity sensing

Previous research has shown that alkali-expanded hydrogen-bonded CLCP coatings can achieve dynamic tunability of broadband reflectance. To achieve such tunability through RH adjustment at room temperature, we utilized a humidity-responsive CLCP thin film material system comprising reactive mesogens (RMs, M1–M4, Fig. S1), photoinitiator Irgacure 651, and right-handed chiral agent R5011 (Fig. S2). Of these, M4 is non-polymerizable and used to swell the CLCP network, which can then be removed via alkaline treatment^45^. Notably, our material system exhibits a temperature-dependent reflection spectrum when uncured, as demonstrated in Fig. S3. Specifically, as the planar-oriented CLC sample is cooled from 85 to 55 °C, the reflection color changes from green to yellow, then to orange, and finally to red. This corresponds to a red shift in the central wavelength of the reflection spectrum from 562 to 718 nm, confirming our texture observations. Upon further cooling, the reflected color gradually shifts towards red and eventually out of the visible range, at which point the CLC mixture in the LC cartridge solidifies and coalesces at 28 °C. Conversely, when the temperature exceeds 85 °C, the CLC reaches its clear point, causing the PBG and the reflected color to disappear, as depicted in Fig. S4. These results indicate that the PBG of CLCP films can be dynamically tuned via thermal control. As a result, the thermochromic properties of hydrogen-bonded CLC substrates offer the possibility of customizing CLCP films to achieve specific reflection bands.

**Fig. 2:**
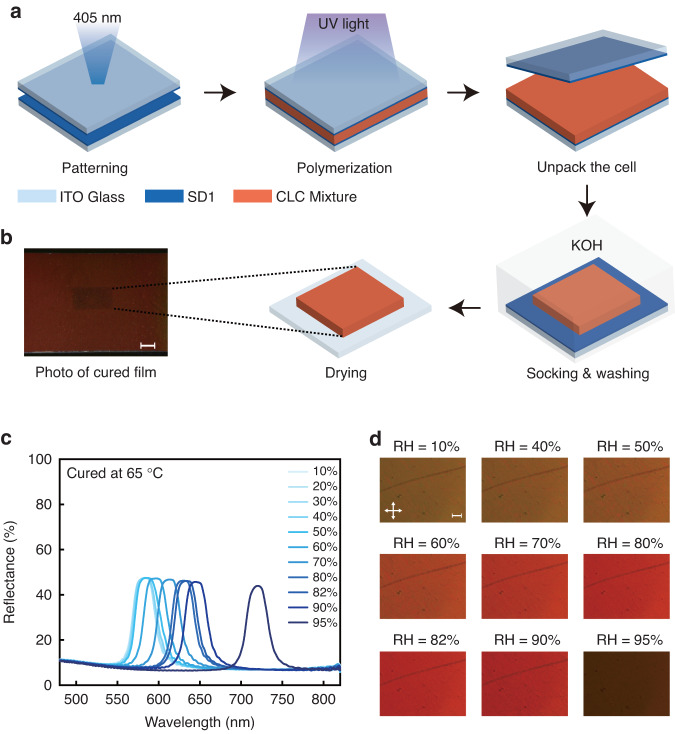
The preparation and reflection spectra of the geometric phase encoded humidity-responsive CLCP films. **a** Fabrication process of humidity-responsive CLCP films with geometric phase encoding. **b** Photo of the films cured at 65 °C. The scale bar is 1 mm. **c** The reflection spectra of humidity-responsive CLCP films (cured at 65 °C) as RH increases from 10% to 95%. **d** The polarizing optical microscope textures of the film exhibit different reflection colors as the RH increases from 10% to 95%. Orthogonal double-arrows indicate crossed polarizers. The scale bar is 100 μm

The process for fabricating humidity-responsive CLCP films encoded with *q*-plate patterns is illustrated in Fig. 2a. Initially, a pattern is recorded on a cell coated with SD1 (Fig. S2) on both inner sides using 405 nm linearly polarized light. This induces the SD1 molecules to align with the direction of the linear polarization of the light through dichroic absorption. Subsequently, the CLC mixture is introduced into the cell in an isotropic state via capillary forces. The patterned control of LC orientation is achieved by transferring the light-induced orderliness to LC molecules through intermolecular forces, in combination with the self-assembly capability of CLC. After polymerization through UV light at an appropriate temperature, the top substrate of the cell, together with the attached CLCP film, is cautiously peeled off and treated with 0.1 M potassium hydroxide (KOH) solution for 15 min. The conversion of carboxylic acids into carboxylate salts after base treatment disrupts the hydrogen bond and forms a polymer salt, enabling a broad tunable range of reflection. Finally, the films are rinsed with pure water and dried using a nitrogen stream, yielding humidity-responsive CLCP films encoded with geometric phase patterns.

We investigated the correlation between PBG and RH in *q*-plate encoded hygroscopic CLCP films synthesized at a temperature of 65 °C (Fig. 2b). The central reflection band of the films was observed to shift from 581 to 719 nm with an increase in RH from 10% to 95% (Fig. 2c). These results reveal the remarkable sensitivity of the CLCP films to RH and demonstrate the potential for tailoring their reflectance properties for various applications. This behavior originates from the disruption of hydrogen bonds in the CLCP film following treatment with an alkaline solution of KOH, leading to the neutralization of carboxylic acid and the formation of carboxylate salts. This enhances the hydrophilicity of the polymer film, ultimately resulting in a hygroscopic CLCP salt^46^. Since polymerization fixes the quantity of cholesteric phase pitch, the film expands upon water absorption, causing an increase in helical pitch and a pronounced redshift in the reflective band. RH serves as a direct indicator of the water content in the environmental conditions. Higher RH values indicate greater moisture in the environment. Consequently, as RH increases, the film absorbs water and expands, elongating the pitch and causing the reflective band to shift towards the red spectrum. Conversely, the opposite occurs as RH decreases. Notably, the PBG remained consistent throughout the shifting process of the reflection band. Figure 2d illustrates the change in structural color at different RH levels. It is worth noting that no significant band shift was observed when RH was less than 40%, possibly due to the decreased dew point at lower RH^47^. For instance, at a room temperature of 25 °C and 40% RH, the dew point temperature is calculated to be 10.47 °C (Eq. (S1)). The substantial difference between the dew point temperature and the room temperature suggests that the air is unsaturated and incapable of condensing the gaseous water into liquid form. Consequently, the film fails to absorb water and expand, leading to negligible movement in the reflective band. The reversibility and repeatability of the humidity-response behavior of these films indicate their stability after two months of storage under ambient conditions. Thus, the relationship between the hygroscopic CLCP films and RH suggests that the PBG can be modulated precisely by humidity.

### Humidity sensing using CLCP films encoded with a single *q*-plate

**Fig. 3:**
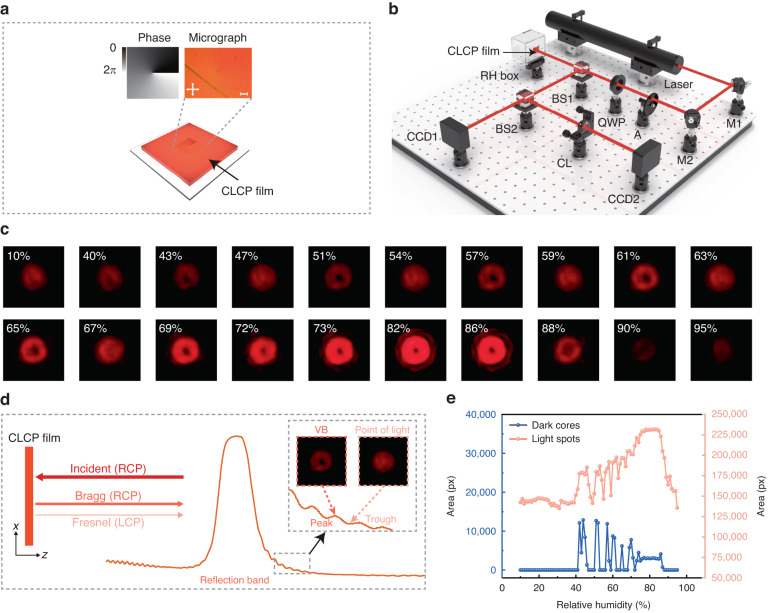
Demonstration of humidity sensing by CLCP films encoded with a single *q*-plate. **a** Schematic representation of a single *q*-plate encoded humidity-responsive CLCP film sample. The illustration shows a geometric phase pattern of the *q*-plate with a topological charge *q* = +0.5 (left), and a micrograph of the encoded positions of humidity-responsive CLCP film cured at 65 °C (right). Orthogonal double-arrows indicate crossed polarizers. The scale bar is 100 μm. **b** Schematic diagram of the single-wavelength humidity monitoring system. Laser, 632.8 nm He–Ne laser, 15 mW/cm²; M mirror, A attenuator, QWP quarter waveplate, BS beam splitter, CL cylindrical lens, CCD charge-coupled device. **c** Reflected diffraction patterns produced by a single *q*-plate encoded humidity-responsive CLCP film as the RH increases from 10% to 95%. **d** Reflected light and diffraction patterns of the CLCP film under RCP light incidence. The thin “Fresnel” arrow refers to incident light reflected at air film interfaces, and the thick arrow labeled “Bragg” refers to circular Bragg reflection. The Bragg reflection at the crest of the reflection band corresponds to the VB, while the Fresnel reflection at the trough corresponds to the light spot. **e** Area of reflected light spots and dark cores at different RHs

The humidity-responsive CLCP film encoded by a single *q*-plate (Fig. 3a) exhibits interesting optical diffraction at the reflection side as a right circularly polarized (RCP) light, which has the same handedness with the CLC helix, impinged perpendicular to the film. To verify the humidity tunability of bandpass OV, a 632.8 nm helium–neon (He–Ne) laser was used as the probe beam to detect the diffraction of a single *q*-plate encoded humidity-responsive CLCP film (Fig. 3b). Herein, the curing temperature of the films was set to 65 °C to provide a wavelength shift of the reflection maximum ranging from 581 to 719 nm, corresponding to a variation of RH between 10% and 95%.

Figure 3c showcases the humidity-dependent modulation of far-field intensity profiles of a VB reflected normally from a single *q*-plate (*q* = +0.5). At RH levels below 40%, no discernible changes were observed in the reflected light spot, with only a faint brightness being apparent. This observation suggests that the probe beam’s wavelength falls outside the PBG of the film. As the RH increases, the presence of reflected light spots singularities becomes noticeable at 40% humidity, indicating the absorption of moisture by the film and subsequent expansion. Within the RH range of 40–86%, the brightness of the diffracted light field progressively intensifies. This phenomenon can be attributed to the film’s pitch increasing as a result of heightened humidity, leading to a red-shift of the reflection band towards the wavelength of the probe beam. Notably, between 73% and 86% humidity, the VB exhibits higher quality due to the reflection band of the film aligning with the wavelength of the probe beam within this humidity range. Once the humidity exceeds 86%, the quality of the VB gradually diminishes, indicating that the wavelength of the probe beam moves out of the PBG of the film and the reflectivity starts to decrease. With further increases in humidity, the diffracted light field merely exhibits light and dark variations of the light spot. Furthermore, the optical response of the film exhibits full reversibility with respect to different humidity levels.

In the RH range of 40–86%, periodic alternations in the light spot reflected by the film are discernible, demonstrating a characteristic pattern of VB and point of light. These observations can be attributed to both the Bragg reflection from the *q*-plate encoded CLCP films and the inescapable Fresnel reflection occurring at the air-film interface^29^ (Fig. 3d). Unlike Bragg reflection, Fresnel reflection does not cause spin angular momentum inversion or geometric phase modulation. In our experiments, a CCD successfully recorded the optical field formed by the superposition of both reflections. Specifically, when the wavelength of the probing beam approached the reflection band peak, the CCD detected the VBs produced by Bragg reflection, due to its higher intensity than Fresnel reflection. Conversely, when the wavelength neared the reflection band trough, the CCD detected a light spot from Fresnel reflection, as its intensity was greater than that of Bragg reflection. To validate this observation, we positioned a left circularly polarized (LCP) filter in front of the CCD, thereby restricting the detection to only the VB, which demonstrated periodic brightness variations in correlation with humidity changes (see Movies S1 and S2 for details). This further ascertained the LCP polarization state of the reflected light spot. This also confirms the property that Bragg and Fresnel reflections have orthogonal polarization states. During the process of raising the RH from 10% to 95% and then down to 10%, we observed a significant and rapid reversible change in the light spot. This remarkable change highlights the excellent moisture absorption and dehydration capabilities of CLCP films and their great potential to develop high-performance real-time humidity sensors (see Movies S3 and S4 for details).

These experimental findings demonstrate that the humidity-responsive CLCP film encoded by a single *q*-plate can generate bandpass VBs with tunable humidity. The resulting OAM modes' topological charge was identified by positioning a cylindrical lens (*f* = 100 mm) in front of the CCD to capture the converted image at the focal plane. The number of dark stripes and tilt direction in Fig. S5 confirmed high-quality OAM states with the expected number (|*m*| = 1, *m* = 2*q*) within the RH range of 73–86%.

To ascertain the viability of visualizing sensing signals, we conducted preprocessing on the recorded reflected light spot using a CCD (Fig. S6). Subsequently, we quantified the area of both the reflected light spot and their corresponding dark cores at various RH levels, as illustrated in Fig. 3e. Intriguingly, we observed consistent and stable areas for the reflected light spots and dark nuclei within the RH range of 79–85%. This finding suggests the generation of high-quality VBs specifically within this RH range. It further substantiates the capability of the CLCP *q*-plate film to convert spectral information, such as wavelength or frequency, into visually discernible light spot images. By scrutinizing the light spot’s morphology, we can accurately determine the corresponding humidity level. Notably, the humidity monitoring system employs a laser as the light source, while the film is affixed to the interior surface without any mechanical contact with the system. Consequently, this system enables remote detection of humidity in confined spaces without requiring direct physical interaction. This unique feature renders it highly suitable for monitoring environments susceptible to flammable and explosive conditions.

### Humidity sensing using CLCP films with four-quadrant *q*-plate array patterns

**Fig. 4:**
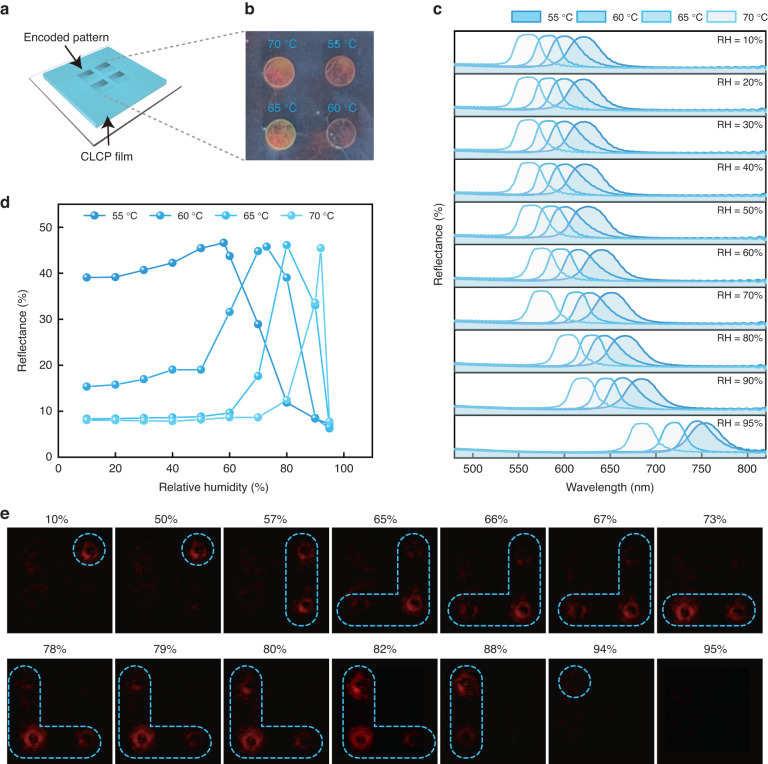
Demonstration of humidity sensing by CLCP films with four-quadrant *q*-plate array patterns. **a** Schematic diagram of a four-quadrant *q*-plate array encoded humidity-responsive CLCP film sample. **b** Photograph of a humidity-responsive CLCP film encoded with a four-quadrant *q*-plate array. **c** Reflection spectra corresponding to each quadrant of the film at different RH. The first, second, third, and fourth quadrants correspond to cured temperatures of 55, 60, 65, and 70 °C, respectively. **d** Reflectance of RCP light (*λ* = 632.8 nm) for each quadrant of the film at different RH. **e** Reflected diffraction patterns of a CLCP film encoded with a four-quadrant q-plate, observed under a single-wavelength humidity monitoring system, as RH increases from 10% to 95%

As previously mentioned, single-wavelength real-time humidity monitoring systems utilize humidity-responsive CLCP films encoded by a single *q*-plate to generate bandpass VBs with adjustable humidity. However, the range of monitored humidity is limited. To address this issue, we expanded the humidity monitoring range by encoding a four-quadrant *q*-plate array pattern onto a CLCP film (Fig. 4a). The samples were locally cured using 405 nm LEDs, following the geometric concept’s quadrant order. The curing temperatures for the first, second, third, and fourth quadrants were 55, 60, 65, and 70 °C, respectively (Fig. 4b). As RH increases from 10% to 95%, the corresponding reflection band shifts in the cured CLCP film at 55 and 70 °C ranges from 620 to 753 nm and from 560 to 684 nm, respectively (Fig. S7). Figure 4c demonstrates the variation of reflection bands corresponding to each quadrant with humidity. Each *q*-plate exhibits a different central wavelength of the reflection band at the same humidity. Furthermore, the reflection bands of the four-quadrant *q*-plate array overlap and undergo a red shift as humidity increases. The reflectance of the films for 632.8 nm RCP light at various relative humidities is depicted in Fig. 4d. The maximum reflectance occurred at 58%, 73%, 82%, and 92% RH for curing temperatures of 55, 60, 65, and 70 °C, respectively. Figure 4c and d demonstrate the alternating display of reflected diffracted light fields from the four-quadrant *q*-plate array pattern encoded CLCP films as humidity changes.

We conducted irradiation on a CLCP film encoded with a four-quadrant *q*-plate array pattern using a collimated, expanded, RCP probe beam with a wavelength of 632.8 nm (Fig. S8). Figure 4e demonstrates that as the RH increases from 10% to 50%, VBs exclusively emerge in the first quadrant, while the light spot remains unchanged. Further increases in RH cause an expansion of the CLCP pitch, leading to a red-shift in the film’s reflection band. Consequently, the reflected VBs are sequentially generated or disappear in accordance with the quadrant order. The above experimental results demonstrate the achievement of a wide range of humidity monitoring through the combination of a single-wavelength humidity monitoring system and the *q*-plate array pattern encoded on the CLCP film. Additionally, we were able to determine the corresponding humidity levels and trends in real-time and in a visualized manner. Notably, the VB pattern in each quadrant has the same variation trend with humidity. The VBs corresponding to the same humidity remain consistent when the humidity increases or decreases, and this process is reversible (Movie S5).

### Humidity sensing using dual-wavelength VBs in CLCP films

**Fig. 5:**
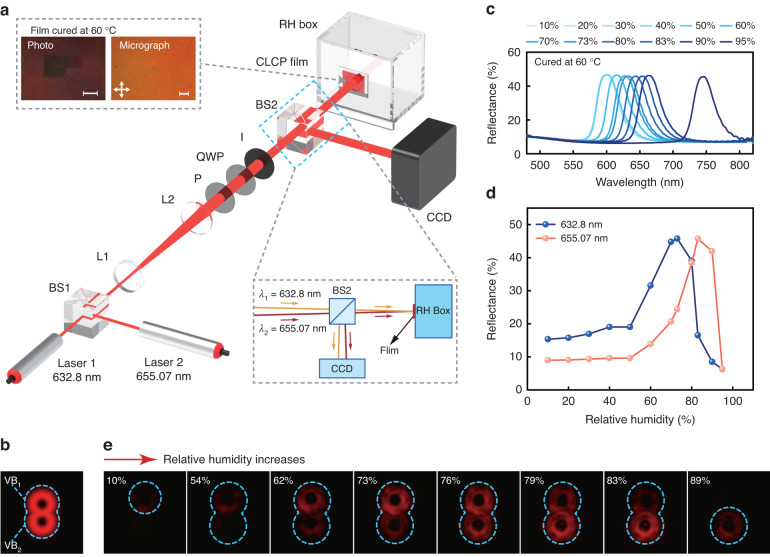
Demonstration of humidity sensing by dual-wavelength VBs in CLCP films. **a** Schematic diagram of the dual-wavelength (Laser 1, 632.8 nm, 10 mW/cm^2^; Laser 2, 655.07 nm, 10 mW/cm^2^) humidity monitoring system. The top left insets show the photograph (scale bar: 1 mm) and micrograph (scale bar: 100 μm) of the humidity-responsive CLCP film encoded with a single *q*-plate (*q* = +0.5) pattern. The film was cured at 60 °C. The bottom right inset is a schematic diagram of the optical path of the double wavelength incident on the surface of the film and reflected. BS beam splitter, L lens, P polarizer, QWP quarter waveplate, I iris, CCD charge-coupled devices. **b** Schematic diagram of the reflected diffraction light field distribution. **c** Reflection spectra of humidity-responsive CLCP films at curing temperatures of 60 °C. **d** Reflectance of the film for two wavelengths (*λ*_1_ = 632.8 nm and *λ*_2_ = 655.07 nm) at different RH. **e** Reflected diffraction patterns of a CLCP film encoded with a single q-plate, observed under a dual-wavelength humidity monitoring system across varying RH levels

As depicted in Fig. 5a, to enable a broad range of humidity monitoring with a single *q*-plate encoded CLCP film, we also developed an alternative dual-wavelength system which employs two laser diodes with wavelengths of 632.8 nm (*λ*_1_) and 655.07 nm (*λ*_2_), separated by ~22 nm. The two laser beams are kept at a certain angle of incidence (inset at the bottom right of Fig. 5a) so that the diffraction light field reflected from the film is distributed as shown in Fig. 5b. This produces two independent visible bands that form a numerical pattern resembling the number "8" with the upper band (VB_1_) corresponding to *λ*_1_ and the lower band (VB_2_) to *λ*_2_.

The CLCP film was cured with UV light at 60 °C and its photograph and micrograph are displayed in the inset at the top left of Fig. 5a. Reflection spectra of the CLCP films at varying RH are shown in Fig. 5c. As the RH increases from 10% to 95%, the reflection band center wavelength shifts from 600 to 744 nm. The region of the reflection bands of the CLCP films cured at this temperature covers the wavelengths of both laser light sources. Figure 5d displays the reflectivity of the films for *λ*_1_ and *λ*_2_ at different RH. The reflectance of both wavelengths slowly increases with humidity below 50% RH, reaching a maximum of *λ*_1_ at 73% RH and *λ*_2_ at 83% RH. As the RH continues to increase, the reflectance of both wavelengths decreases gradually. At 95% RH, the reflectance of both wavelengths drops to its lowest value. Notably, the reflectance curves of the two wavelengths intersect at 76% RH, indicating the equivalent brightness of the two VBs.

Figure 5e shows the variation of the reflected diffraction patterns of the film observed by the dual-wavelength humidity monitoring system. When the RH is below 54%, VB_1_ gradually becomes brighter than VB_2_. In the RH range of 54–83%, the pattern similar to the numeric character "8" formed by two VB donuts gradually appears from top to bottom as the RH increases. At 76% RH, the pattern "8" is clearly visible. As the RH continues to increase, only VB_2_ can be observed at 89% RH, and the pattern "8" disappears when the RH is >95%. The gradual appearance and disappearance of the pattern "8" from top to bottom enables the trend of ambient humidity to be judged. Overall, the results demonstrate that the combination of the single *q*-plate encoded CLCP film and inexpensive laser diodes in the humidity monitoring system is capable of achieving an extended range of RH monitoring in real-time and visually (Movie S6).

## Discussion

Stimuli-responsive LC polymers have been developed to respond to different stimuli signals. However, their sensing mechanisms rely on either naked-eye perceptibility of structural color or measurement of wavelength changes by spectrometric tools, which limit their practical applications due to a trade-off between accuracy and commercialization, hindering their widespread use in daily life. To address this issue, we propose a novel sensing scheme that utilizes geometric phase-encoded stimuli-responsive CLCPs to autonomously detect environmental humidity while simultaneously providing an immediate visual pattern. Our approach demonstrates a unique sensing mechanism since a stimulus signal causes changes in the structure of the encoded CLCP film, which, in turn, leads to corresponding changes in the shape of the reflected diffraction light field under a geometric phase light beam. The use of a highly directional laser light source enables long-range sensing measurements without the need for physical contact. This study highlights the potential of this approach for creating cost-effective and visually intuitive sensing systems, which has not been achieved before using geometric phase-encoded stimuli-responsive CLCP films.

In this study, we employed *q*-plates to encode stimuli-responsive CLCPs, generating recognizable “donut” patterns for remote, contactless humidity monitoring. What is noteworthy is that our proposed new sensing approach can utilize not only VBs but also a variety of beam types such as Bessel beams and Airy beams. These beams possess non-diffractive properties and self-healing capabilities, allowing for long-distance transmission while also enabling diversification and customization of visual patterns. Furthermore, integrating machine learning techniques with these patterned visual sensing signals promises the realization of more sophisticated liquid crystal optical sensing technologies. Beyond humidity measurement, our scheme holds potential for varied applications. Its non-contact sensing ability is advantageous in environments where mitigating interaction or potential harm to the measurement subject is necessary. Moreover, the customization of CLCP properties enables simultaneous multi-parameter measurements of humidity, temperature, and light intensity—practical in complex fields like agriculture, chemicals, and environment monitoring^13^. Additionally, our approach can be extended to fiber-optic end-face sensing, allowing direct environmental parameter measurement along transmission lines by affixing a CLCP film to the fiber’s end-face—a potential asset in communication and energy transmission systems^48^.

Some limitations of the system in our study should be addressed. For example, the humidity response of the CLCP film is limited due to the lower dew point at lower RH, requiring further optimization for humidity monitoring below this range. Additionally, complete immersion of the film in an alkaline solution can cause the photoalignment agent to dissolve, resulting in film detachment from the glass substrate and subsequent degradation of the reflected diffraction patterns. Therefore, in future research, alternative patterning methods will be explored to improve the quality and durability of the diffraction patterns.

In conclusion, our proposed sensing scheme utilizes geometric phase-encoded stimuli-responsive CLCP films for real-time remote monitoring. The proof of concept demonstrated the detection of distinguishable circular patterns of reflective OVs generated by *q*-plate encoded stimuli-responsive CLCP films by detecting the additional DoF of light, providing an alternative to traditional and often costly spectroscopic measurements. The four-quadrant *q*-plate array CLCP film and the integrated dual-wavelength VB were designed to sense humidity trends and extend the humidity monitoring range. Additionally, the high directionality of the laser light source enables contactless and long-range humidity measurements with spatial specificity. In essence, recognizable pattern changes can be generated from any geometric phase-encoded CLCs with reflective bands that respond to external stimuli. We anticipate that this approach will expand the application of the LC-mediated geometric phase and facilitate the commercialization of stimuli-responsive LCs as optical sensors for environmental monitoring and other applications.

## Materials and methods

### Materials

The CLC mixture mainly consists of 96.86% LC reactive monomers (HCCH, China), 2.64% chiral dopant (R5011, HCCH, China), and 0.5% photoinitiator (Irgacure 651, TCI, Japan). The LC monomers comprise M1–M4 (see their respective chemical structures in Fig. S1) at a weight ratio of 15:30:30:15. The chemical structure of R5011 and Irgacure 651 can be found in Fig. S2. The helical twisting power (HTP) of R5011 is ~108 μm^−1^. The mixture is magnetically stirred at 1000 rpm at 70 °C for 30 min to ensure complete blending. Besides, the alignment agent SD1 (BJRC, China, see its chemical structure in Fig. S2) is dissolved in N,N-dimethylformamide (DMF) with a weight concentration of 0.3%. The SD1 solution is then filtered through a 0.2-μm Teflon syringe filter to remove any impurities. KOH was obtained from Aladdin.

### Photopatterned process

The indium-tin-oxide (ITO) glass substrates (20 mm × 25 mm) were initially cleaned using ultrasonic treatment and subsequently exposed to oxygen plasma for 10 min. Following this, a photoalignment layer was formed by spin-coating the SD1 solution onto the substrate at 800 rpm for 10 s, followed by 3000 rpm for 40 s. The coated substrate was then cured at 100 °C for 10 min. Two SD1-coated substrates were subsequently joined together using 20 μm-thick double-sided adhesive tapes to create a cell.

To achieve the desired director distributions of either a single *q*-plate or *q*-plate arrays, the empty cell was exposed to a polarization-controllable dynamic photopatterning system utilizing a digital micromirror device (DMD). The surface director distribution is calculated for each pattern and divided into 18 sub-regions spanning from 0° to 170° at 10° intervals. Each subregion is endowed with a uniform director value, from *π*/18 to *π* in intervals of *π*/18. The exposure time for each subregion is 180 s, with a light power of 4.38 mW/cm². Each subregion is exposed while simultaneously rotating the polarizer by 10°. This exposure process enables the desired director distribution to be achieved on the substrate. Subsequently, the right-handed CLC mixture is introduced into the photopatterned LC cassette through capillary action at 120 °C, followed by UV light curing at an appropriate temperature.

### Measurement

The reflection micrograph of the sample was observed using a metallurgical microscope (BA210MET, Motic, China), and the reflectance spectrum was recorded using a fiber optic spectrometer (USB4000, Ocean Optics, USA) paired with a halogen tungsten light source. Reflected diffraction light spots were captured by a CCD camera (E3ISPM, TOUPTEK, China). The RH was regulated using a humidity generator (FD-HG, Furande, China).

### Supplementary information

Supplementary Information

Movie S1_The VBs at Increasing RH after Filtering Left-Circular Polarized Light

Movie S2_The VBs at Decreasing RH after Filtering Left-Circular Polarized Light

Movie S3_The VBs at Decreasing RH

Movie S4_The VBs at Increasing RH

Movie S5_The Four-Quadrant VB Array at Increasing RH

Movie S6_The Dual-Wavelength VBs at Increasing RH

## Data Availability

The data that support the findings of this study are available from the corresponding author upon reasonable request.
