# Supplementary material for: Geometric phase-encoded stimuli-responsive cholesteric liquid crystals for visualizing real-time remote monitoring: humidity sensing as a proof of concept
Source: Light Sci Appl. 2024 Jan 24;13:27. doi: 10.1038/s41377-023-01360-7 (PMC10805905; doi:10.1038/s41377-023-01360-7)
Supplement: Supplementary file 1 — Supplementary Information [file 41377_2023_1360_MOESM1_ESM.docx]

**Supplementary Information**

**Geometric phase-encoded stimuli-responsive cholesteric liquid crystals for visualizing real-time remote monitoring: humidity sensing as a proof of concept**

Shi-Long Li^1^, Zhao-Yi Chen^1^, Peng Chen^2^, Wei Hu^2^, Chaohong Huang^1,3^, Sen-Sen Li^1,3^, Xuejia Hu^1,3^, Yan-Qing Lu^2^* and Lu-Jian Chen^1,3^*

^1^Department of Electronic Engineering, School of Electronic Science and Engineering, Xiamen University, Xiamen 361005, China

^2^College of Engineering and Applied Sciences, Nanjing University, Nanjing 210093, China

^3^Fujian Key Laboratory of Ultrafast Laser Technology and Applications, Xiamen University, Xiamen, 361005, China

***Correspondence:** Yan-Qing Lu (yqlu@nju.edu.cn) or Lu-Jian Chen (lujianchen@xmu.edu.cn)


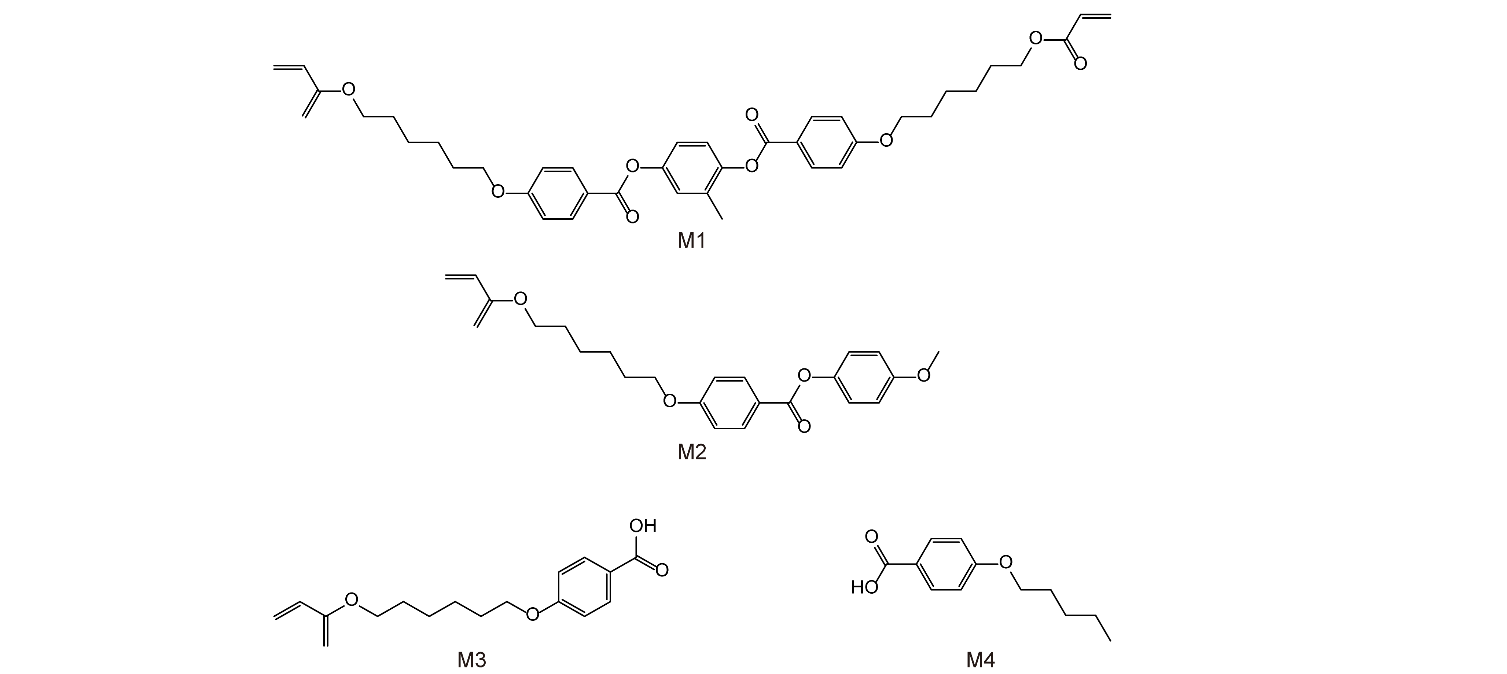


**Fig. S1** Molecule structures of the utilized reactive monomers.


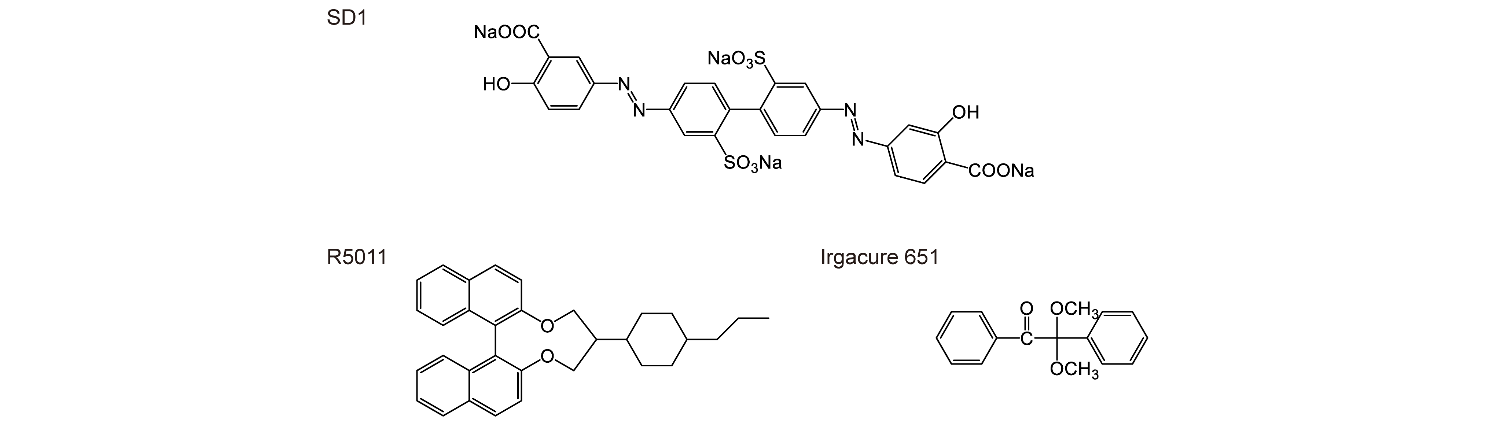


**Fig. S2** Molecule structures of the photoalignment agent SD1, right-handed chiral dopant R5011 and photoinitiator Irgacure 651.


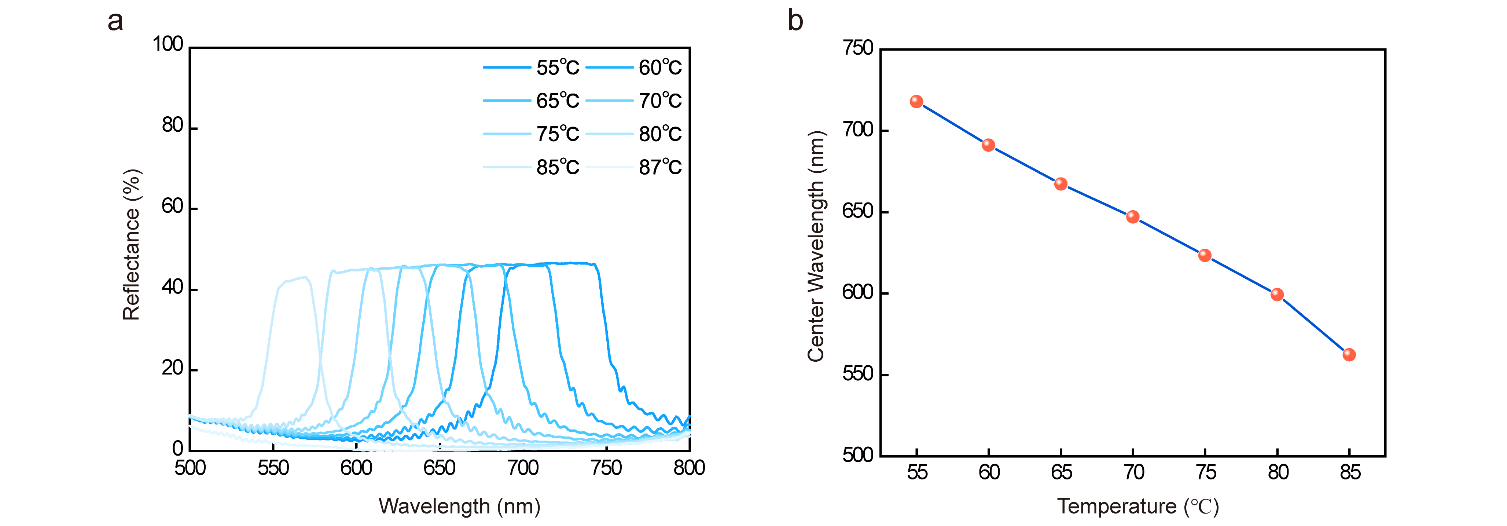


**Fig. S3 a** Reflection spectra and **b** reflection band center wavelength at different temperatures (55℃, 60℃, 65℃, 70℃, 75℃, 80℃, 85℃, 87℃) for the uncured CLC mixture.


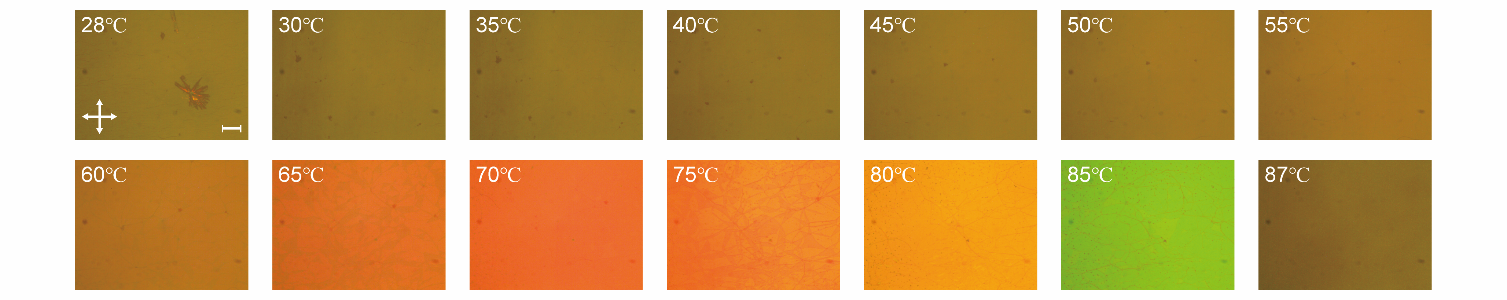


**Fig. S4** Reflection micrographs of uncured CLC mixture at different temperatures. The scale bar is 100 μm.

| $t_{\text{d}}=\frac{B*\left(\ln\left(\frac{RH}{100}\right)+\frac{A*t}{B+t}\right)}{A-\ln\left(\frac{RH}{100}\right)-\frac{A*t}{B+t}}$ |
| --- |

**Eq. S1** The extended Magnus equation is used for the relationship between the dewpoint and the relative humidity and temperature. The *t*_d_ is the temperature of the dewpoint (°C), A and B are coefficients, RH is the relative humidity (%) and t is the temperature (°C)^S1^. The coefficients are obtained from literature, A = 17.625 and B = 243.04°C^S2^.


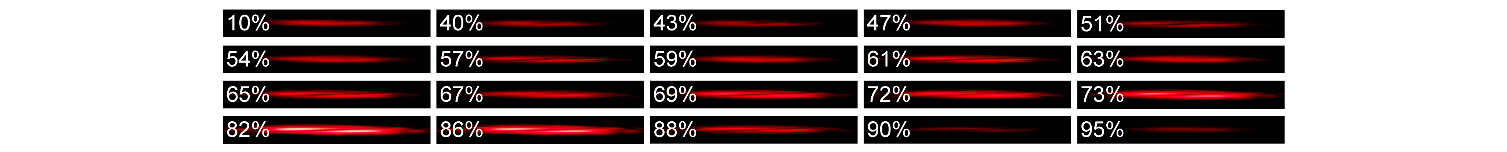


**Fig. S5** Topological charge detection photograph produced by a single q-plate encoded humidity-responsive CLCP film as the RH increases from 10% to 95%.


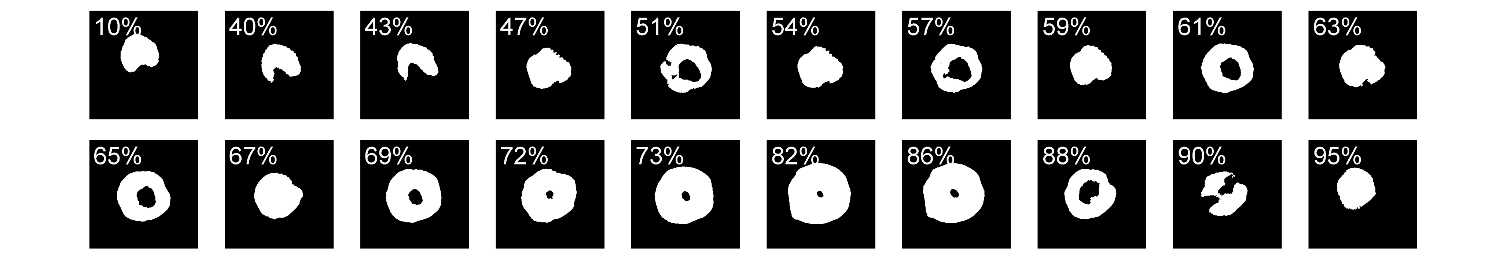


**Fig. S6** Binary diagram of the reflected diffraction light spot at different humidity levels processed with MATLAB.


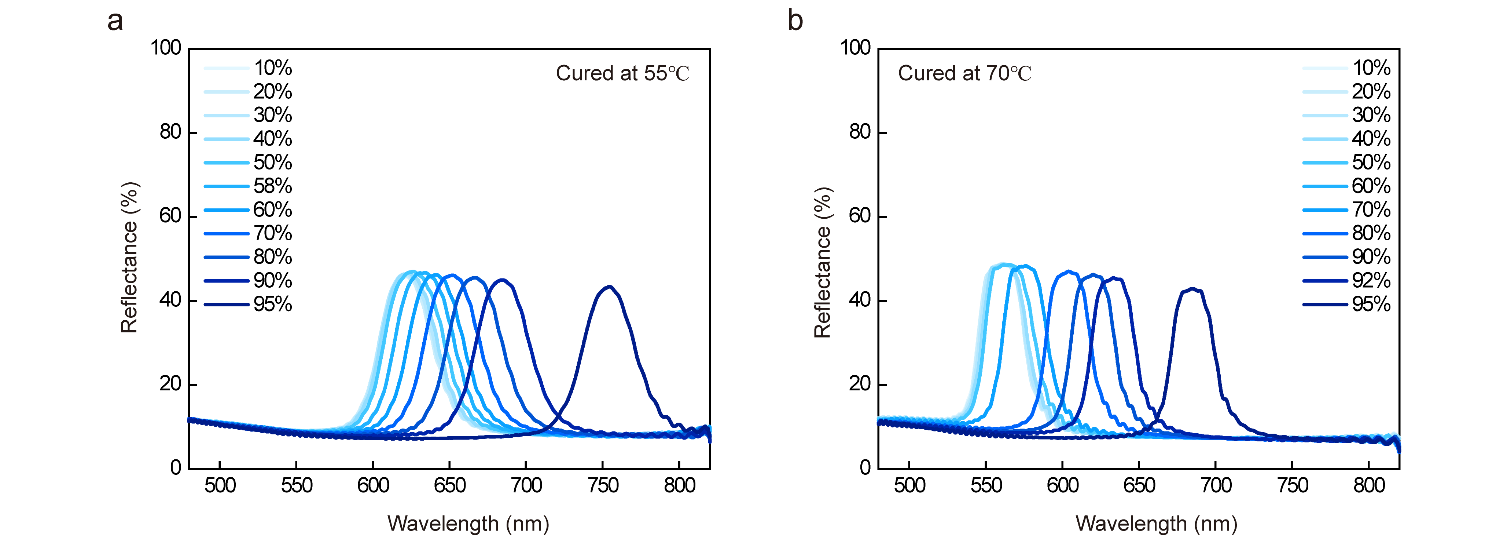


**Fig. S7** Reflection spectra of humidity-responsive CLCP films at curing temperatures of **a** 55℃ and **b** 70℃, respectively.


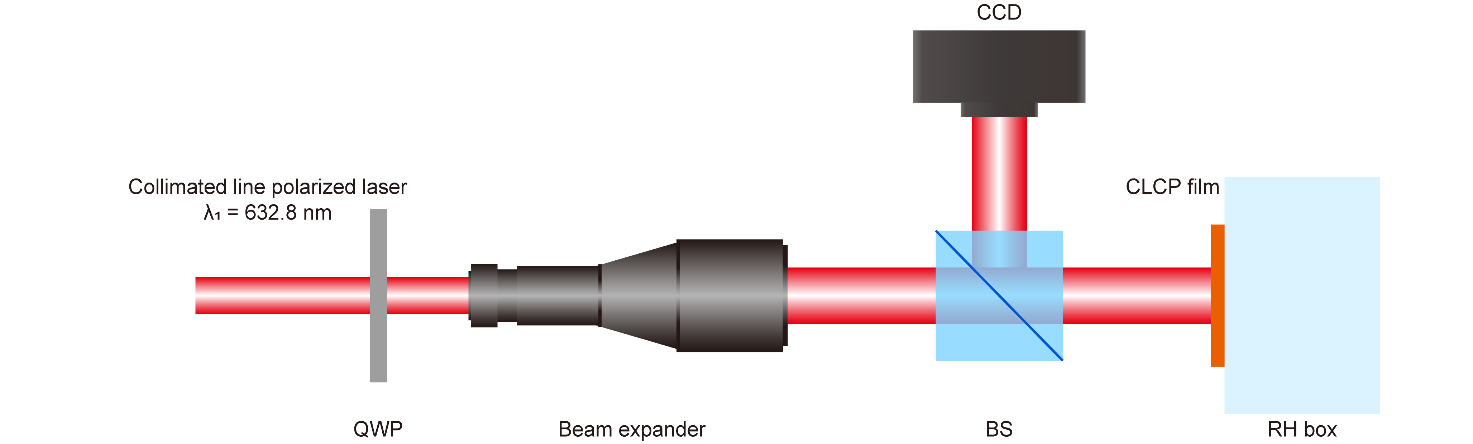


**Fig. S8** Schematic diagram of the single-wavelength humidity monitoring system with beam expander system. QWP quarter waveplate, BS beam splitter, CCD charge-coupled devices.

**References**

S1 Lawrence, M. G. The relationship between relative humidity and the dewpoint temperature in moist air - A simple conversion and applications. *Bull*. *Amer*. *Meteorol*. *Soc*. **86,** 225–234 (2005).

S2 Alduchov, O. A. & Eskridge, R. E. Improved magnus form approximation of saturation vapor pressure. *J*. *Appl*. *Meteorol*. **35,** 601–609 (1996).
